# Telemedicine follow-up for pre-malignant and malignant glottic lesions: a randomised controlled trial study protocol comparing care close to home versus standard of care

**DOI:** 10.2340/1651-226X.2024.43947

**Published:** 2025-08-12

**Authors:** Nathalie F. van Rhee, Rosanne C. Schoonbeek, Inge Wegner, Karin M. Vermeulen, Robert C. Maat, Dirk A. Dietz de Loos, György B. Halmos, Boudewijn E.C. Plaat

**Affiliations:** a Department of Otorhinolaryngology – Head and Neck Surgery, University Medical Center Groningen, University of Groningen, Groningen, The Netherlands; b Graduate School of Medical Sciences, University of Groningen, Groningen, The Netherlands; c Department of Epidemiology, University Medical Center Groningen, University of Groningen, Groningen, The Netherlands; d Department of Otorhinolaryngology, Saxenburgh Medical Center, Hardenberg, The Netherlands; e Department of Otorhinolaryngology, Isala Hospital, Zwolle, The Netherlands

**Keywords:** Head and neck cancer, telemedicine, follow-up, patient-reported outcome measures, quality of life, patient satisfaction, clinical trial protocol

## Abstract

**Background and purpose:**

In the Netherlands, care for head and neck cancer (HNC) is centralised in head and neck oncology centres (HNOCs). Follow-up after treatment requires frequent visits that can burden patients and providers. Telemedicine, through remote evaluation of laryngopharyngoscopy videos recorded at local hospitals, may offer a feasible alternative. This study protocol describes the aim to assess patient satisfaction and safety with telemedicine follow-up after treatment of (pre-)malignant glottic lesions, including severe dysplasia, carcinoma-in-situ and T1 squamous cell carcinoma, conducted at one HNOC and participating general hospitals.

**Methods and analysis:**

As a non-blinded, randomised controlled trial, 90 patients with a one-way travel time by car of over 45 min to the HNOC will be allocated to the intervention group (follow-up by an Ear, Nose and Throat surgeon at a nearby participating hospital) or the control group (standard follow-up at the HNOC). All patients undergo guideline-based care. In the intervention group, laryngopharyngoscopy recordings will be remotely reviewed by HNOC specialists on the same day. Surveys will be fulfilled at baseline, 6 and 12 months. The primary outcome is overall patient satisfaction using a 0–10 numeric rating scale at 12 months follow-up. Secondary outcomes are safety, quality of life, fear of recurrence, travel time and carbon-dioxide emission. Safety will be assessed through recurrence, complications, re-referral and survival. Between-group and within-group comparisons will be performed to evaluate differences in outcomes, using appropriate statistical methods based on data distribution.

**Ethics and dissemination:**

This study explores regional collaboration and sustainable follow-up for HNC patients. The ethics board approved the protocol (M23.325004). The authors commit to publishing the findings.

**Trial registration number:**

ClinicalTrials.gov ID NCT06940505.

## Introduction

Globally, head and neck cancer (HNC) is the 7th most common cancer diagnosis [1]. The most common primary sites include carcinomas of the oral cavity, pharynx and larynx. In recent years, the overall incidence of HNC in the Netherlands has increased, mainly due to the rise in human papilloma virus-related squamous cell carcinoma of the oropharynx [2, 3].

Aiming to detect cancer without delay and identify recurrences early, patients follow a rigorous post-treatment schedule. In the Netherlands, follow-up for HNC patients is centralised in head and neck oncology centres (HNOCs) [4]. Dutch guidelines advise visits every 2 to 3 months during the first 2 years, and every 4 to 6 months in the following 3 years [4]. This applies to all HNC patients, despite significant differences in recurrence rates and prognosis [2]. Centralised care requires some patients to travel long distances, creating financial and practical burdens for them and their caregivers [5, 6]. With a growing patient population, head and neck oncologists also face challenges in maintaining oncologic (follow-up) care. There are several efforts to address rising costs and burden, advocating for digital innovations, regional cooperation and sustainable solutions [7].

The need to revise the patient-care pathway is also internationally recognised and extends beyond HNC to other oncological fields [8–12]. One proposed solution is patient-initiated follow-up, where patients schedule consultations based on symptoms, abandoning a generalised schedule [8, 13]. However, this may be less sustainable for older or frail patients. Evidence that routine follow-up for (head and neck) cancer improves clinical outcomes is generally lacking [14, 15]. An exception may be early-stage glottic carcinoma after transoral laser surgery (TOLS), for which reported recurrence rates vary widely, ranging from 9 to 25% depending on the tumour stage and follow-up duration [16–19]. Early detection may allow limited revision surgery instead of escalating to multimodality treatment with radiotherapy [14, 15, 17, 20]. Patients also report that follow-up reduces fear of recurrence and provides psychosocial support [12]. Follow-up for HNC usually includes high definition (HD)-laryngoscopy, which has a high detection rate for malignancies, and adding narrow-band-imaging (NBI) improves detection even further [16, 21].

Building upon the idea of regional cooperation whilst making use of technological advancements, follow-up by an Ear, Nose and Throat (ENT) surgeon at a nearby hospital combined with remote evaluation of laryngoscopy videos (telemedicine) may offer a practical solution. Telemedicine follow-up addresses the burden of travel, which may also reduce carbon-dioxide (CO_2_) emissions [22].

Therefore, a study was primarily designed to assess patient satisfaction with telemedicine follow-up after TOLS for pre-malignant lesions and early-stage glottic carcinoma (severe dysplasia, carcinoma-in-situ and T1 squamous cell carcinoma), using patient-reported outcome measures (PROMs) and patient-reported experience measures (PREMs). To the best of our knowledge, no comparable previous studies have been performed before. PROMS and PREMs are widely used standardised tools to assess patients’ health status and experiences with care from their own perspective. PROMs assess health outcomes like symptom burden and quality of life, whilst PREMs focus on aspects such as communication, accessibility and overall satisfaction [23, 24]. Moreover, we aim to evaluate if both follow-up techniques are equally safe in terms of detection of disease recurrence, complications, re-referral and survival.

## Methods and analysis

### Study setting

This non-blinded, randomised controlled trial (RCT) is carried out at the Department of Otorhinolaryngology, Head and Neck Surgery of the University Medical Center Groningen (UMCG), a HNOC and academic tertiary referral hospital in the Netherlands. The participating general hospitals are Isala Medical Centre and Saxenburgh hospital. The Dutch HNC patient association (Patiëntenvereniging Hoofd-Hals, PVHH) provided valuable input into the development of the study design. This protocol is reported according to the SPIRIT guideline (Supplementary information A) [25].

### Eligibility criteria

**Figure 1:**
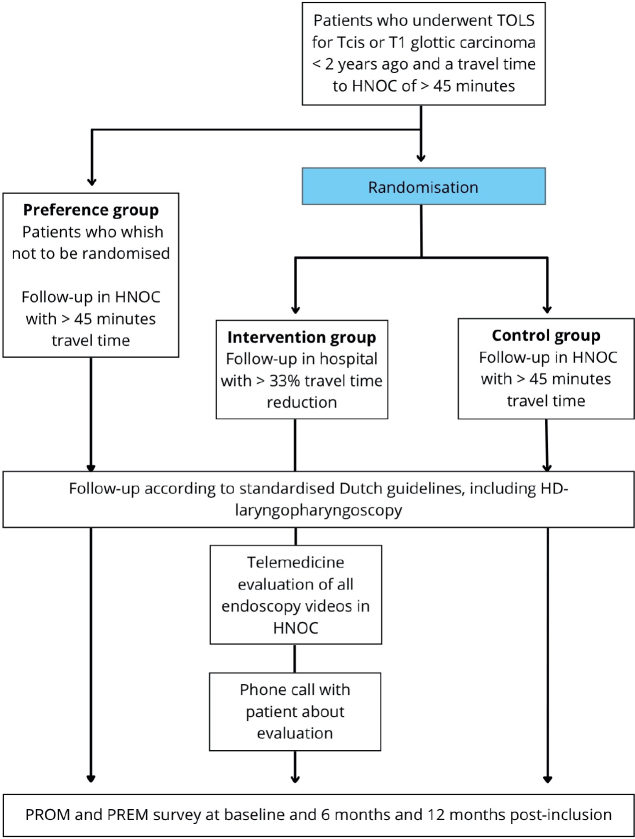
Inclusion and follow-up protocol. TOLS: transoral laser surgery; TCIS: carcinoma in-situ; HNOC: head and neck oncology centre; PROM: patient-reported outcome measure; PREM: patient-reported experience measure.

Inclusion criteria include patients who underwent TOLS for severe dysplasia, carcinoma-in-situ or T1 squamous cell carcinoma, have a one-way travel time (by car) to the HNOC of > 45 min and have the possibility of follow-up in a nearby participating hospital. In addition, patients are eligible for inclusion if they are within 2 years postoperatively and can speak and write Dutch (Figure 1). Patients will be excluded if they continue to undergo follow-up for other cancers in the HNOC. Written informed consent will be obtained from all patients (Supplementary information B).

### Interventions

Figure 1 visualises the study flow. Included patients will be randomly assigned to one of two groups: an intervention group and a control group. Follow-up is performed though standardised follow-up guidelines and is equal for all patients, with at least of 4 to 6 consultations expected [4]. The control group will receive their oncological follow-up in the UMCG. The intervention group will receive their oncological follow-up by an ENT surgeon in a hospital that is closer to the patients’ home. A reduction in travel time of more than one-third was considered indicative of closer proximity to home, as it is believed that it would be a significant time reduction for the patients. A history will be taken, and a complete ENT physical examination will be performed, including flexible HD-video laryngopharyngoscopy (with or without NBI) and neck palpation. HD-laryngopharyngoscopy is performed with digital transnasal flexible endoscopes of XION (XION GmbH, Berlin, Germany) or Olympus (Olympus Nederland BV). The videos obtained during the follow-up visit will be digitally transferred and securely stored in the electronic patient files in the HNOC, for the same day assessment by a head and neck oncologist. Furthermore, the head and neck oncologist has access to the patients’ previous examinations, (surgical) history and findings or concerns of the ENT surgeon who has seen the patient earlier that day. After evaluation, the patient is contacted by the HNOC to share the results, and a new management plan (such as re-referral to the HNOC) is formulated if necessary.

Patients who do not wish to participate in this randomised controlled study will be asked to complete the questionnaires that are part of this study whilst receiving their follow-up in HNOC (the ‘preference group’).

### Outcomes

Our primary outcome is satisfaction with care and is measured on a 0–10 numeric rating scale (NRS) at 12 months post-inclusion. Our secondary outcome is safety, which is evaluated and measured by recurrence, re-referral rate, complications following recurrence treatment and survival. For the secondary outcomes, patients will be followed for at least 1 year after inclusion. Safety outcomes are only assessed after the study termination, thereby aiming for at least 2 years follow-up per patient. All outcome measures are outlined in Table 1.

**Table 1:** Outcome measures.

Outcome measure	Assessment	Evaluation method	Timing of evaluation
**Patient satisfaction (PREMs)**			
Overall satisfaction with care	0–10 NRS	Survey	Baseline, at 6 and 12 months
**Satisfaction with travel time**	0–10 NRS	Survey	Baseline, at 6 and 12 months
**Specific satisfaction**	Questions about satisfaction with care from CQI questionnaires [33, 35]	Survey	Baseline, at 6 and 12 months
**Safety**			
Local and regional disease recurrence	In case of recurrence: date of diagnosis, clinical and pathological characteristics, date and type of treatment, time until treatment to assess for delay	Electronic patient file	End of follow-up period
**Complications post-recurrence treatment**	After treatment in case of recurrence: frequency of complications and type of complications	Electronic patient file	End of follow-up period
**Re-referral rate**	Frequency of re-referral of intervention group patients to HNOC	Electronic patient file	End of follow-up period
**Disease-specific and overall survival**	Overall mortality and mortality related to (recurrent) disease	Electronic patient file	End of follow-up period
**PROMs**			
HRQoL	EQ-5D-5L questionnaire, assesses five dimensions of health: mobility (1), self-care (2), usual activities (3), pain/discomfort (4), and anxiety/depression (5) [31, 32]. Each dimension is rated on a 5-level scale, ranging from no problems (1) to extreme problems (5). Additionally, the EQ-5D-5L includes include a 0–100 VAS.	Survey	Baseline, at 6 and 12 months
**Symptom burden**	Four subdomains of the EORTC QLQ-H&N-35 questionnaire (weight loss, speech, swallowing function and pain) [30]. The H&N35 employs a 4-point response scale (‘not at all’ to ‘very much’), with higher scores indicating a higher symptom burden.	Survey	Baseline, at 6 and 12 months
**Fear of recurrence**	CWS, 6 items on worries after cancer treatment rated on a 4-point scale from almost never/ not at all to almost always/ very much [34].	Survey	Baseline, at 6 and 12 months
**Other**			
Visit frequency	Amount of visits (scheduled and extra visits)	Electronic patient file	End of follow up period
**Travel distance**	Total distance travelled (kilometres) from the patient’s home address to the hospital by car	As indicated by Google Maps	End of follow up period
**Travel time**	Subjectively reported travel time, for the patients and their care givers	Survey	Baseline and at 6 months
**CO_2_ emission**	Estimation of CO_2_ emission caused by travel to the hospital for follow-up	Calculated from the travel distance (kilometres) and the mean emission per kilometre [36]	End of follow up period

Abbreviations: PREMs: patient-reported experience measures; NRS: numeric rating scale; CQI: consumer quality index; PROMs: patient-reported outcome-measures; HRQoL: health-related quality-of-life; VAS: visual analogue scale; CWS: cancer worry scale; EORTC QLQ-H&N-35: European Organisation for Research and Treatment of Cancer Quality of Life Questionnaire Head and Neck Module 35; CO_2_: carbon-dioxide.

### Sample size and recruitment

A power analysis was performed to calculate the sample size needed to answer our primary research question. As telemedicine follow-up has not been studied in HNC patients, power analysis was based on data from non-oncologic populations [26–28]. Most of these studies are one-armed studies. These studies reported a mean patient satisfaction, measured with a NRS or VAS, of 8.6 to 8.8, with one study noting a baseline score of 8.2. Although these results may not be representative of the HNC population, we based our expectations with regard to the primary outcome on these studies, nonetheless. Patient satisfaction is estimated at 9 (standard deviation [SD] 1.8) in the intervention arm and 8 (SD 1.8) in the control arm. To detect a clinically relevant difference of 1 point with 80% power and a two-sided alpha of 0.05, at least 41 patients per group are required. With an expected drop-out of 10%, a total of 90 patients will be included. This analysis was performed using G*Power (version 3, Heinrich-Heine-Universität Düsseldorf, Germany). Patients are first informed about the study by their treating head and neck oncologist in the HNOC. If a patient meets the eligibility criteria, the researcher will provide additional information, and an informed consent will be obtained.

### Allocation to study groups

An independent researcher will generate the randomisation chart to ensure allocation concealment for the patient, clinician and researcher. Randomisation will be performed in REDCap with a 1:1 allocation ratio. Block randomisation and stratification will not be used, as no baseline characteristics are expected to significantly influence the study outcomes. Blinding of the treatment allocation after randomisation is not feasible, as both patients and care providers will be aware of the hospital setting in which the follow-up takes place.

### Data collection methods

Baseline characteristics collected from electronic patient files include age, gender, clinical and histological tumour data, (surgical) history and comorbidities. Comorbidities are graded using the Adult Comorbidity Evaluation (ACE-27) as none, mild, moderate or severe [29]. Follow-up data will be collected via patient files or surveys and stored in REDCap (Table 1). Only the researchers have access to this securely stored data.

Patients will complete a 39-question survey at baseline (Supplementary information C) and again at 6 and 12 months. The survey includes validated and new questions, developed with support from a clinical epidemiologist (KV) with expertise in questionnaires (Table 1) [30–35].

The baseline survey is completed on paper after inclusion but before randomisation. A shorter version is used during follow-up. Intervention group patients receive surveys at baseline or by mail and complete them during a scheduled call with researcher assistance if required. Patients followed at the HNOC complete surveys after follow-up visits, also with help if required.

### Statistical analysis

Descriptive statistics will be used to summarise patient’s baseline characteristics. Categorical variables will be presented as absolute numbers and percentages of the total. Continuous variables are tested for normality using Kolmogorov-Smirnov analysis and will be presented as means and SDs for normally distributed data, and medians and interquartile ranges for non-normally distributed data. Between-group mean (or median) differences, rate differences and rate ratios with 95% confidence intervals (or ranges) will be calculated. The groups will be compared using the Wilcoxon signed rank test or the Mann-Whitney test for non-normally distributed data, the independent t-test for normally distributed data and chi-squared test for categorical variables. Additionally, within-group changes will be assessed with paired t-tests (continuous data) and the McNemar test (categorical data). Kaplan-Meier survival analysis will be performed for disease-specific and overall survival. The EQ-5D-5L, EORTC QLQ-H&N-35 and cancer worry scale (CWS) questionnaires will be analysed according to their manual [30–32, 34]. Total CO_2_ emission caused by travel to the hospital will be estimated and compared between groups based on the values provided by the European Environment Agency for the period 2010–2023 (130.1 g of CO_2_ per kilometre) [36]. Missing values will be handled using multiple imputation, assuming that there is only missingness at random, and all analyses will be performed on an intention-to-treat basis. The data will be reported according to the CONSORT guidelines [37].

## Discussion and conclusion

This is the first prospective study investigating the novel concept of relocating follow-up care for HNC patients. Since the COVID-19 pandemic, telemedicine use has notably increased and is widely accepted by both oncologic and non-oncologic patients [26–28, 38]. In most telemedicine studies in otolaryngology, videoconferencing consultations are used, with remote physical examinations occasionally employed for otoscopic evaluations but not for laryngoscopic (follow-up) assessments [39, 40]. Advancements in HD-laryngoscopy and emerging Artificial Intelligence (AI) disease detection software may create opportunities to reorganise follow-up care for HNC patients, reducing dependency on physician expertise [41, 42].

The strength of the study is its RCT design and the inclusion of a preference arm. This study aligns with sustainability goals. The possibility of telemedicine follow-up offers a more flexible and patient-centred approach to care, reducing travel burden and increasing accessibility. It may also help alleviate pressure on specialised centres by enabling efficient, remote monitoring without compromising safety. A limitation of the study is the use of a non-validated survey. However, most questions are derived from validated questionnaires, and the benefit of validation of the survey was not seen. Additionally, patients in the intervention group will complete the survey in a different setting than those in the control group (i.e. at home with remote assistance through a telephone call and at the HNOC with assistance in person as needed), which may introduce bias. With the knowledge that a follow-up duration of 12 months is limited, safety outcomes will only be assessed after the conclusion of the study period. Furthermore, travel distances to hospitals in the Netherlands are typically shorter than in other countries; however, the findings of this study may also be applicable to larger countries, where they could potentially benefit an even greater number of patients. If patients are satisfied and safety is confirmed, future research should explore other patient groups suitable for telemedicine follow-up. Multicentre studies will be necessary to assess its national impact and inform guideline changes, benefiting patients, society and the environment.

## Supplementary Material



## Data Availability

Upon request, the (pseudonymised) dataset can be made available after conclusion of the study period. The authors commit to publishing the study’s results.
