# Supplementary material for: Telemedicine follow-up for pre-malignant and malignant glottic lesions: a randomised controlled trial study protocol comparing care close to home versus standard of care
Source: Acta Oncol. 2025 Aug 12;64:43947. doi: 10.2340/1651-226X.2024.43947 (PMC13064437; doi:10.2340/1651-226X.2024.43947)
Supplement: Supplementary file 1 [file AO-64-43947-s1.pdf]

SUPPLEMENTARY INFORMATION A, SPIRIT CHECKLIST

SPIRIT

STANDARD PROTOCOL ITEMS: RECOMMENDATIONS FOR INTERVENTIONAL TRIALS

SPIRIT 2013 Checklist: Recommended items to address in a clinical trial protocol and related documents*

Section/item	Item No	Description	Addressed on page number
**Administrative information**			
Title	1	Descriptive title identifying the study design, population, interventions, and, if applicable, trial acronym	1
Trial registration	2a	Trial identifier and registry name. If not yet registered, name of intended registry	2
	2b	All items from the World Health Organization Trial Registration Data Set	Yes, page 12 ClinicalTrials.gov ID NCT06940505
Protocol version	3	Date and version identifier	N/A
Funding	4	Sources and types of financial, material, and other support	11
Roles and responsibilities	5a	Names, affiliations, and roles of protocol contributors	12
	5b	Name and contact information for the trial sponsor	N/A
	5c	Role of study sponsor and funders, if any, in study design; collection, management, analysis, and interpretation of data; writing of the report; and the decision to submit the report for publication, including whether they will have ultimate authority over any of these activities	12

	5d	Composition, roles, and responsibilities of the coordinating centre, steering committee, endpoint adjudication committee, data management team, and other individuals or groups overseeing the trial, if applicable (see Item 21a for data monitoring committee)	N/A
**Introduction**			
Background and rationale	6a	Description of research question and justification for undertaking the trial, including summary of relevant studies (published and unpublished) examining benefits and harms for each intervention	5, 6
	6b	Explanation for choice of comparators	5, 6
Objectives	7	Specific objectives or hypotheses	6
Trial design	8	Description of trial design including type of trial (eg, parallel group, crossover, factorial, single group), allocation ratio, and framework (eg, superiority, equivalence, noninferiority, exploratory)	7, 9
**Methods: Participants, interventions, and outcomes**			
Study setting	9	Description of study settings (eg, community clinic, academic hospital) and list of countries where data will be collected. Reference to where list of study sites can be obtained	6
Eligibility criteria	10	Inclusion and exclusion criteria for participants. If applicable, eligibility criteria for study centres and individuals who will perform the interventions (eg, surgeons, psychotherapists)	7
Interventions	11a	Interventions for each group with sufficient detail to allow replication, including how and when they will be administered	7, 8
	11b	Criteria for discontinuing or modifying allocated interventions for a given trial participant (eg, drug dose change in response to harms, participant request, or improving/worsening disease)	12
	11c	Strategies to improve adherence to intervention protocols, and any procedures for monitoring adherence (eg, drug tablet return, laboratory tests)	9
	11d	Relevant concomitant care and interventions that are permitted or prohibited during the trial	N/A

Outcomes	12	Primary, secondary, and other outcomes, including the specific measurement variable (eg, systolic blood pressure), analysis metric (eg, change from baseline, final value, time to event), method of aggregation (eg, median, proportion), and time point for each outcome. Explanation of the clinical relevance of chosen efficacy and harm outcomes is strongly recommended	8, Table 1
Participant timeline	13	Time schedule of enrolment, interventions (including any run-ins and washouts), assessments, and visits for participants. A schematic diagram is highly recommended (see Figure)	7, Figure 1
Sample size	14	Estimated number of participants needed to achieve study objectives and how it was determined, including clinical and statistical assumptions supporting any sample size calculations	8
Recruitment	15	Strategies for achieving adequate participant enrolment to reach target sample size	N/A

Methods: Assignment of interventions (for controlled trials)

Allocation:			
Sequence generation	16a	Method of generating the allocation sequence (eg, computer-generated random numbers), and list of any factors for stratification. To reduce predictability of a random sequence, details of any planned restriction (eg, blocking) should be provided in a separate document that is unavailable to those who enrol participants or assign interventions	9
Allocation concealment mechanism	16b	Mechanism of implementing the allocation sequence (eg, central telephone; sequentially numbered, opaque, sealed envelopes), describing any steps to conceal the sequence until interventions are assigned	9
Implementation	16c	Who will generate the allocation sequence, who will enrol participants, and who will assign participants to interventions	9
Blinding (masking)	17a	Who will be blinded after assignment to interventions (eg, trial participants, care providers, outcome assessors, data analysts), and how	9
	17b	If blinded, circumstances under which unblinding is permissible, and procedure for revealing a participant's allocated intervention during the trial	9

Methods: Data collection, management, and analysis

Data collection methods	18a	Plans for assessment and collection of outcome, baseline, and other trial data, including any related processes to promote data quality (eg, duplicate measurements, training of assessors) and a description of study instruments (eg, questionnaires, laboratory tests) along with their reliability and validity, if known. Reference to where data collection forms can be found, if not in the protocol	9
	18b	Plans to promote participant retention and complete follow-up, including list of any outcome data to be collected for participants who discontinue or deviate from intervention protocols	9, 10
Data management	19	Plans for data entry, coding, security, and storage, including any related processes to promote data quality (eg, double data entry; range checks for data values). Reference to where details of data management procedures can be found, if not in the protocol	9
Statistical methods	20a	Statistical methods for analysing primary and secondary outcomes. Reference to where other details of the statistical analysis plan can be found, if not in the protocol	9, 10
	20b	Methods for any additional analyses (eg, subgroup and adjusted analyses)	10
	20c	Definition of analysis population relating to protocol non-adherence (eg, as randomised analysis), and any statistical methods to handle missing data (eg, multiple imputation)	10
**Methods: Monitoring**			
Data monitoring	21a	Composition of data monitoring committee (DMC); summary of its role and reporting structure; statement of whether it is independent from the sponsor and competing interests; and reference to where further details about its charter can be found, if not in the protocol. Alternatively, an explanation of why a DMC is not needed	N/A
	21b	Description of any interim analyses and stopping guidelines, including who will have access to these interim results and make the final decision to terminate the trial	N/A
Harms	22	Plans for collecting, assessing, reporting, and managing solicited and spontaneously reported adverse events and other unintended effects of trial interventions or trial conduct	N/A
Auditing	23	Frequency and procedures for auditing trial conduct, if any, and whether the process will be independent from investigators and the sponsor	N/A
**Ethics and dissemination**			

Research ethics approval	24	Plans for seeking research ethics committee/institutional review board (REC/IRB) approval	12
Protocol amendments	25	Plans for communicating important protocol modifications (eg, changes to eligibility criteria, outcomes, analyses) to relevant parties (eg, investigators, REC/IRBs, trial participants, trial registries, journals, regulators)	12
Consent or assent	26a	Who will obtain informed consent or assent from potential trial participants or authorised surrogates, and how (see Item 32)	8
	26b	Additional consent provisions for collection and use of participant data and biological specimens in ancillary studies, if applicable	N/A
Confidentiality	27	How personal information about potential and enrolled participants will be collected, shared, and maintained in order to protect confidentiality before, during, and after the trial	9
Declaration of interests	28	Financial and other competing interests for principal investigators for the overall trial and each study site	11
Access to data	29	Statement of who will have access to the final trial dataset, and disclosure of contractual agreements that limit such access for investigators	9
Ancillary and post-trial care	30	Provisions, if any, for ancillary and post-trial care, and for compensation to those who suffer harm from trial participation	N/A
Dissemination policy	31a	Plans for investigators and sponsor to communicate trial results to participants, healthcare professionals, the public, and other relevant groups (eg, via publication, reporting in results databases, or other data sharing arrangements), including any publication restrictions	12
	31b	Authorship eligibility guidelines and any intended use of professional writers	12
	31c	Plans, if any, for granting public access to the full protocol, participant-level dataset, and statistical code	12
**Appendices**			
Informed consent materials	32	Model consent form and other related documentation given to participants and authorised surrogates	Supplementary information B

Biological specimens	33	Plans for collection, laboratory evaluation, and storage of biological specimens for genetic or molecular analysis in the current trial and for future use in ancillary studies, if applicable	N/A

*It is strongly recommended that this checklist be read in conjunction with the SPIRIT 2013 Explanation & Elaboration for important clarification on the items. Amendments to the protocol should be tracked and dated. The SPIRIT checklist is copyrighted by the SPIRIT Group under the Creative Commons "Attribution-NonCommercial-NoDerivs 3.0 Unported" license.

SUPPLEMENTARY INFORMATION B: Information for Participation in Research

Research into New Diagnostics for Head and Neck Cancer
Information about the development and application of Artificial Intelligence for the detection of head and neck cancer: towards more sustainable care close to the patient (PaNaMa ID 17695)

Introduction

Dear Sir/Madam,

We are asking you to participate in a scientific study. Participation is voluntary. However, your (written) consent is required to take part.

At the University Medical Center Groningen (UMCG), medical-scientific research is being conducted. This research may aim to find better methods to diagnose or treat a disease, or to gain more insight into bodily functions and disease processes.

Before you decide whether to participate, this letter provides you with an explanation of what the study entails. Please read the information carefully and feel free to ask the researcher any questions. Contact details are listed at the end of this letter. You may also discuss your participation with your partner, family, or friends.

This research is being conducted by the Department of Otolaryngology / Head and Neck Oncology at the UMCG. The Central Ethics Review Committee of the UMCG has assessed the study to ensure it complies with current Dutch legislation and additional UMCG regulations.

1. Background, purpose, and study design

You are regularly seen for follow-up after treatment for laryngeal cancer. Normally, these check-ups are performed at the UMCG. With this study, we aim to investigate the impact of travel distance on patient satisfaction, satisfaction and quality of life. If you live far from the UMCG, your check-ups could also be carried out by an ENT specialist closer to home, after which the information and digital images are securely sent to the UMCG. These images are then assessed by the head and neck oncologist at the UMCG. On the same day as your local ENT appointment, a staff member from the UMCG Head and Neck Oncology Center will contact you with the final results of the check-up. If you or your treating physician wishes or deems it necessary, you will be seen again at the UMCG.

To compare patients with different travel distances, you will either continue to be seen at the UMCG or have follow-up care closer to home (if possible). Group assignment is random. Patients who live close to the UMCG can also participate as a control group.

Images from the flexible endoscopy performed on you (showing the inside of the throat and larynx, including the vocal cords) are stored as photos and videos. These are necessary for communicating your results, but we also intend to use them for scientific research. Specifically, we aim to develop a reliable

computer program that can automatically recognize abnormalities in the vocal cords. In the future, such a program could assist doctors using artificial intelligence (AI).

2. What participation involves and what we expect from you

To assess your health and satisfaction, you will be asked to fill out a few questionnaires. You don't need to do anything else for the study except give permission for the use of the data/images collected during your care. Along with the images, we will collect information about your diagnosis, age, and risk factors for throat abnormalities (such as smoking).

We are asking for your consent to use this information.

If you need time to think it over, you can submit the signed consent form later at the ENT outpatient clinic desk or bring it during your next visit.

3. Possible benefits and risks

It's important to weigh the potential benefits and risks before deciding. If you're assigned to the group with shorter travel distance, this could save you time and money. Your treatment changes in that your follow-up will take place at a local hospital and you'll receive a call with the results by the end of the day. You will not undergo any additional or different throat examinations. Similarly, those who continue their follow-up at the UMCG will not receive new treatments or different exams.

4. If you do not want to participate or wish to withdraw

You decide whether to participate and consent to the use of the images. You may withdraw at any time, without giving a reason. Just let the researcher know, either verbally or in writing. You don't need to complete any additional forms. Data collected up to that point will still be used in the study.

5. Collection, use, and storage of your data

Your data will be collected, used, and stored on secure computers at the UMCG. Each participant receives a code attached to their medical data.

Confidentiality of your data

To protect your privacy, your data will be coded. Your name and other directly identifying details will be removed. The researcher and research team will know which code belongs to you. Only with the code key can your identity be traced. This key is securely stored at the UMCG. The data and images sent to external parties who help develop and improve the computer program contain only the code and are not traceable to you. Likewise, in reports and publications about the study, your data will not be identifiable.

Access to your data for monitoring

Certain individuals may access all your data at the research site, including uncoded data, to verify that the study is being conducted properly. This may include authorized monitors, auditors, or regulatory

authorities such as the Dutch Health and Youth Care Inspectorate. They are required to keep your information confidential. We ask for your consent for this access.

Data retention period

By law, the researcher must retain your collected data for 15 years for the purpose of this study. After that, the data will be destroyed.

More information about your privacy rights can be found in the UMCG's privacy statement: http://uwprivacy.umcg.nl. By signing the consent form, you give permission for the collection, retention, and use of your medical and personal data as described in this letter.

You will not be personally informed of the project's outcomes.

Use of data for future research

We are requesting permission to store your data for future related research. This will always be for studies in the same field that build on or complement this study.

6. Compensation for participation

There is no compensation for participation in this study.

7. Do You have questions?

If you have questions, please contact the researcher N.F. van Rhee.

If you have complaints about the study, you can discuss them with the researcher or your treating physician. If you prefer to speak to someone not involved in the study, you can contact the UMCG complaints officer.

For questions or complaints about how your personal data is handled, we recommend contacting the Data Protection Officer of the UMCG.
Phone: 050-361 61 61
Email: privacy@umcg.nl

Kind regards,
Drs. N.F. van Rhee, Physician-Researcher

On behalf of:
Dr. B.E.C. Plaat, ENT specialist / Head and Neck Oncologic Surgeon

Contact Details

Drs. N.F. van Rhee
Physician-Researcher, ENT Department, University Medical Center Groningen

Email: AI@kno.umcg.nl

Dr. B.E.C. Plaat
ENT Specialist / Head and Neck Oncologic Surgeon
ENT Department, University Medical Center Groningen
P.O. Box 30 001, Hanzeplein 1, 9700 RB Groningen
Phone: 050-3612898

Attachment: Participant Consent Form

SUPPLEMENTARY INFORMATION C, Baseline survey (translated from Dutch)

Patient Survey: Experiences with Head and Neck Cancer Follow-up Appointments

Telemedicine Follow-Up for Early Laryngeal Cancer: a randomized controlled trial study comparing care close to home versus standard of care

To assess your health and satisfaction, we have asked you to complete this questionnaire. The researcher present will assist you if needed. Please answer the questions honestly; your responses will remain confidential and will not be shared with your doctor. Therefore, do not write any personally identifiable information on this form, such as your name or date of birth

The questionnaire begins on the next page.

Health questionnaire

Under each heading, please choose the ONE answer that best describes your health TODAY.

Question 1. MOBILITY

- I have no problems in walking about ☐
- I have slight problems in walking about ☐
- I have moderate problems in walking about ☐
- I have severe problems in walking about ☐
- I am unable to walk about ☐

Question 2. SELF-CARE

- I have no problems washing or dressing myself ☐
- I have slight problems washing or dressing myself ☐
- I have moderate problems washing or dressing myself ☐
- I have severe problems washing or dressing myself ☐
- I am unable to wash or dress myself ☐

Question 3. USUAL ACTIVITIES (e.g. work, study, housework, family or leisure activities)

- I have no problems doing my usual activities ☐
- I have slight problems doing my usual activities ☐
- I have moderate problems doing my usual activities ☐
- I have severe problems doing my usual activities ☐
- I am unable to do my usual activities ☐

Question 4. PAIN / DISCOMFORT

- I have no pain or discomfort ☐
- I have slight pain or discomfort ☐
- I have moderate pain or discomfort ☐
- I have severe pain or discomfort ☐
- I have extreme pain or discomfort ☐

Question 5. ANXIETY / DEPRESSION

- I am not anxious or depressed ☐
- I am slightly anxious or depressed ☐
- I am moderately anxious or depressed ☐
- I am severely anxious or depressed ☐
- I am extremely anxious or depressed ☐

Question 6.

We would like to know how good or bad your health is TODAY.

This line is numbered from 0 to 100.

100 means the <u>best</u> health you can imagine.
0 means the <u>worst</u> health you can imagine.

Please mark an X on the line to show how your health is TODAY.

Now, write the number you marked on the line in the box below.

YOUR HEALTH TODAY = [ ]

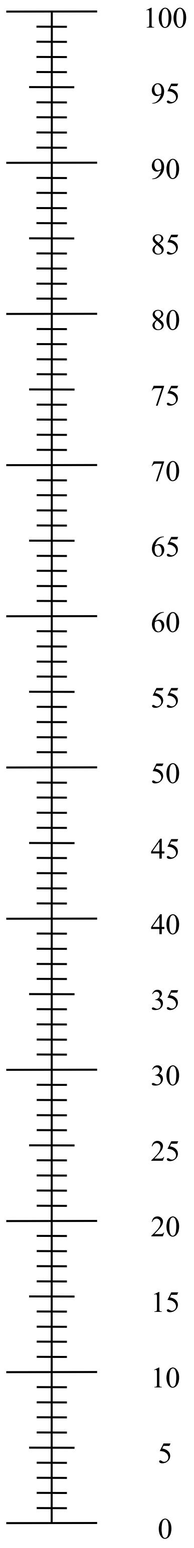

The worst health
you can imagine

Head and Neck Region Symptoms

Patients sometimes report that they have the following symptoms or problems. Please indicate the extent to which you have experienced these symptoms or problems during the past week. Please answer by circling the number that best applies to you.

During the past week:

	Not at all	A little	Quite a bit	Very much
**Question 7.** Have you had problems swallowing liquids	1	2	3	4
**Question 8.** Have you had problems swallowing pureed food?	1	2	3	4
**Question 9.** Have you had problems swallowing solid food?	1	2	3	4
**Question 10.** Have you choked when swallowing?	1	2	3	4
**Question 11.** Have you been hoarse?	1	2	3	4
**Question 12.** Have you had trouble talking to other people?	1	2	3	4
**Question 13.** Have you had trouble talking on the telephone?	1	2	3	4
**Question 14.** Have you had a painful throat?	1	2	3	4
			No	Yes
**Question 15**. Have you lost weight?			1	2

Worry/ concern

The next questions are about whether you have been worried about cancer. For each question, can you please check the answer that best reflects how you have felt **during the past week?**

Question 16. How often have you thought about your chance of getting cancer (again)?

Almost never ❑
Sometimes ❑
Often ❑
Almost always ❑

Question 17. How often have your thoughts about the chance of getting cancer (again) affected your mood?

Almost never ❑
Sometimes ❑
Often ❑
Almost always ❑

Question 18. How often have your thoughts about the chance of getting cancer (again) made it difficult for you to carry out your daily activities?

Almost never ❑
Sometimes ❑
Often ❑
Almost always ❑

Question 19. How worried are you about the possibility of getting cancer (again) someday?

Not at all ❑
A little ❑
Quite a bit ❑
Very much ❑

Question 20. How often have you worried about your chance of getting cancer (again)?

- Almost never ❑
- Sometimes ❑
- Often ❑
- Almost always ❑

Question 21. To what extent is this worry a problem for you?

- Not at all ❑
- A little ❑
- Quite a bit ❑
- Very much ❑

Follow-up appointments in the hospital

The following questions are about your appointments with the doctor **in the past 6 months**. It concerns the doctor who checks how you are doing after your cancer treatment.

Question 22. Did the doctor take you seriously?

- Never ❑
- Sometimes ❑
- Usually ❑
- Always ❑

Question 23. Did the doctor listen to you attentively?

- Never ❑
- Sometimes ❑
- Usually ❑
- Always ❑

Question 24. Did the doctor have enough time for you?

- Never ❑
- Sometimes ❑
- Usually ❑
- Always ❑

Question 25. Did you have confidence in the doctor's expertise?

Never ☐
Sometimes ☐
Usually ☐
Always ☐

Question 26. Was the doctor aware of your file/situation?

No, not at all ☐
A little ☐
Largely ☐
Yes, completely ☐
I don’t know (anymore) ☐

Question 27. Did the doctor explain things to you in an understandable way?

Never ☐
Sometimes ☐
Usually ☐
Always ☐

Question 28. Could you ask the questions you wanted?

Never ☐
Sometimes ☐
Usually ☐
Always ☐

Question 29. On a scale from 0 to 10, where 0 is 'completely dissatisfied' and 10 is 'completely satisfied,' how satisfied are you with the care you receive from your doctor? Circle your answer.

0 1 2 3 4 5 6 7 8 9 10

Employment status

Question 30. How many hours per week do you work? Only count the hours for which you are paid.

……… hours

Travel time and transportation

The following questions are about your experience with your travel and transportation to the follow-up appointments **in the past 6 months**. Check what applies to you.

Question 31. Was it a problem to get to your hospital? Consider the accessibility with your own transportation, public transport, or taxi.

A big problem ❑
A small problem ❑
No problem ❑

Question 32. How do you usually travel to a follow-up appointment?

By car ❑
By taxi ❑
By public transport ❑
Walking/ by bike ❑
In another way, namely ……………………………………………………………. ❑

Question 33. If you come by car, what do you think of the parking fees at your hospital?

A big problem ❑
A small problem ❑
No problem ❑
Not applicable ❑

Question 34. How many people do you usually travel with to a follow-up appointment, besides yourself?

No others ❑
1 extra person ❑
2 extra persons ❑
More than 2 extra persons ❑

Question 35. How much time do you spend in total (from the moment you leave until you are home again to go to a follow-up appointment?

……….. hours and ……… minutes

Question 36. How much time do you spend traveling for the one-way trip to the hospital?

……….. hours and ……… minutes

Question 37. If you travel to the follow-up appointment with other people: how much time does this person spend traveling for the one-way trip (including the time they spend getting to you)? If you go to your appointments alone, please skip this question.

……….. hours and ……… minutes

Question 38. Do you often combine your hospital visit with another appointment? You can check multiple options.

No ❑

Yes, I sometimes or often combine this with another hospital appointment ❑

Yes, I sometimes or often combine this with another (social) private appointment ❑

Question 39. On a scale of 0 to 10, where 0 is 'completely dissatisfied' and 10 is 'completely satisfied,' how satisfied are you with your travel time to your follow-up appointments? Circle your answer.

0 1 2 3 4 5 6 7 8 9 10

<u>This was the end of the questionnaire.</u>
